# Maternal Immune Thrombocytopenia With Prior Splenectomy as a Cause of Severe Neonatal Thrombocytopenia: A Case Report

**DOI:** 10.7759/cureus.107983

**Published:** 2026-04-29

**Authors:** Sanae Kheir, Mohammed Ech-Chebab, Anass Ayyad, Sahar Messaoudi, Rim Amrani

**Affiliations:** 1 Pediatrics, Mohammed VI University Hospital, Oujda, MAR; 2 Neonatology and Neonatal Intensive Care, Mohammed VI University Hospital, Oujda, MAR; 3 Neonatal Intensive Care Unit, Mohammed VI University Hospital, Mohammed I University, Oujda, MAR; 4 Neonatology, Faculty of Medicine and Pharmacy, Mohammed VI University Hospital, Mother and Child Health Laboratory, Oujda, MAR

**Keywords:** hemorrhagic syndrome, immune thrombocytopenic purpura, neonatal thrombocytopenia, newborn, splenectomized mother

## Abstract

Severe neonatal thrombocytopenia is a formidable complication of maternal immune thrombocytopenic purpura (ITP). We report the case of a male newborn, preterm at 32 weeks of gestation, presenting on day 4 of life with jaundice and severe cutaneous hemorrhagic syndrome. The maternal history was significant for severe ITP requiring splenectomy. The initial assessment showed a significant drop in platelets, with a count of 6,000/mm³, even though the bone marrow aspirate appeared normal. Treatment included corticosteroids, platelet transfusions, polyvalent immunoglobulins, and phototherapy. This approach led to a gradual improvement, and the platelet count returned to normal within three months. This case highlights the association between the severity of maternal ITP and the risk of severe neonatal thrombocytopenia. It also emphasizes the importance of a thorough, team-based treatment plan that combines treatments to relieve symptoms with immunomodulatory therapies.

## Introduction

Neonatal thrombocytopenia is a common blood disorder that affects newborns. It is defined as having a platelet count of less than 150,000/mm³. In neonatal intensive care units, the rate of this disorder is between 20% and 30% [1,2]. Neonatal thrombocytopenia occurs in approximately 10-30% of infants born to mothers with immune thrombocytopenia [3]. Severe neonatal thrombocytopenia (platelet count <50×10⁹/L) occurs in approximately 10-15% of cases, with an estimated risk of intracranial hemorrhage of 1-3% [3],[4]. We report a case illustrating the complex management of a newborn with severe thrombocytopenia associated with significant jaundice, occurring in the context of severe maternal ITP requiring splenectomy.

## Case presentation

**Figure 1:**
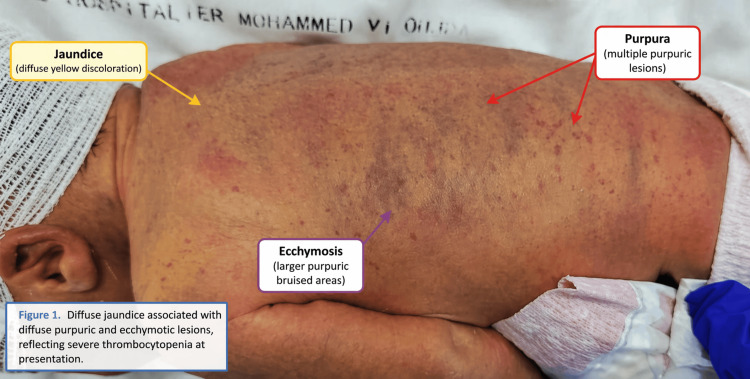
Diffuse jaundice associated with extensive purpuric and ecchymotic lesions, reflecting severe thrombocytopenia at presentation.

We report the observation of a male newborn admitted on day 4 of life for neonatal jaundice and hemorrhagic syndrome. He was the product of a 32-week gestation, born vaginally with a weight of 2000 g. The mother, aged 23, is being monitored for immune thrombocytopenic purpura (ITP) and underwent a splenectomy at age 17. The maternal complete blood count at delivery indicated normal hemoglobin and white blood cell levels accompanied by isolated thrombocytopenia (platelet count: 47,000/mm³), indicative of severe thrombocytopenia. Upon admission, the newborn presented with cutaneous-mucosal jaundice and diffuse purpuric and ecchymotic lesions (Figure 1).

**Figure 2:**
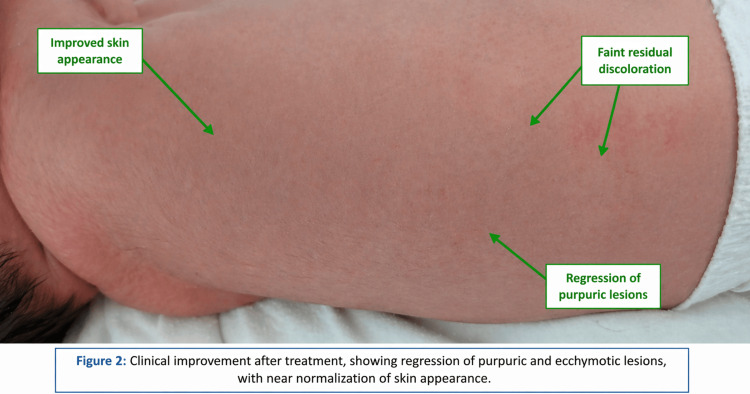
Marked regression of purpuric and ecchymotic lesions at day 7 of life following treatment, indicating clinical improvement.

The initial biological assessment revealed severe thrombocytopenia at 6000 platelets/mm³, confirmed on a citrated tube (5500/mm³). The myelogram performed was normal, indicating a peripheral origin. Management included oral corticosteroid therapy and a transfusion of irradiated platelets, allowing a transient rise to 28,000/mm³, followed by the administration of polyvalent immunoglobulins (two doses of 2 g) with an improvement in the platelet count to 46,000/mm³. A transfusion of red blood cell concentrates was subsequently necessary for anemia. Phototherapy was administered for neonatal jaundice. The cutaneous hemorrhagic manifestations regressed in less than one week under treatment (Figure 2).

**Figure 3:**
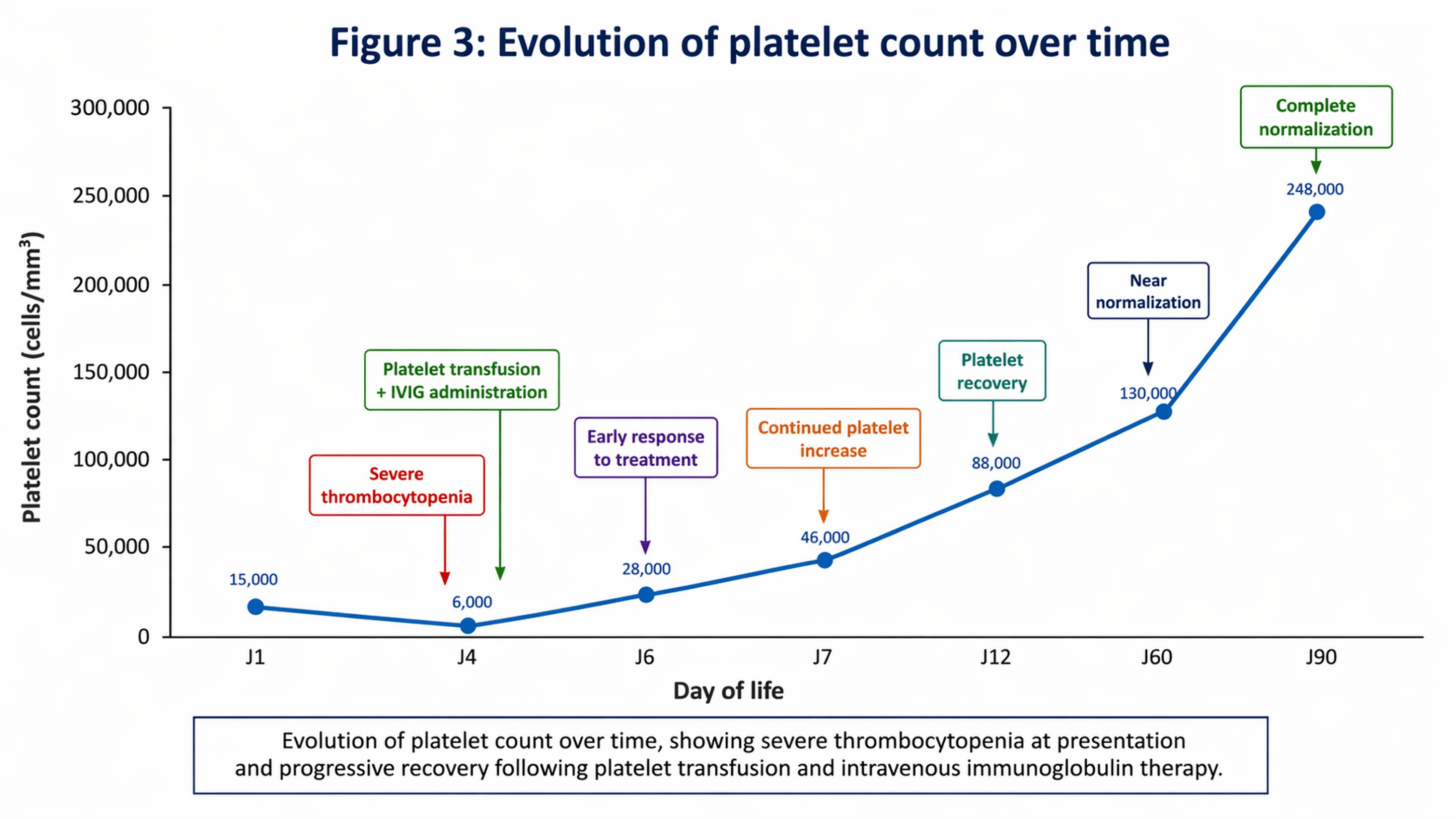
Platelet count progression showing severe thrombocytopenia at onset and gradual normalization after treatment.

Thrombocytopenia, on the other hand, took longer to correct, with a count of 88,000/mm³ on day 12, then 130,000/mm³ at two months, before complete normalization to 248,000/mm³ at three months of age (Figure 3). A summary of laboratory findings over time is presented in Table 1.

**Table 1:** Chronological evolution of laboratory parameters.

Day of life	Platelet count (/mm³)	Hemoglobin (g/dL)	WBC (/mm³)	Neutrophils (/mm³)
Day 1	15,000	17.7	21,890	10,288
Day 4	6,000	12.9	9,330	2,610
Day 6	28,000	10.6	20,080	8,630
Day 7	46,000	10.3	12,950	5,580
Day 12	88,000	13.7	17,880	8,290
Day 60	130,000	13.4	9,800	1,980
Day 90	248,000	13.0	9,900	2,000

## Discussion

Neonatal thrombocytopenia secondary to maternal ITP results from the transplacental transfer of maternal IgG antiplatelet antibodies, leading to increased peripheral platelet destruction in the fetus and neonate [5].

Several studies have suggested that severe maternal ITP and a history of splenectomy may be associated with a higher risk of neonatal thrombocytopenia. However, available data remain inconsistent, and maternal platelet count or disease severity does not reliably predict neonatal platelet levels [6,7]. In our case, the presence of severe maternal disease requiring splenectomy supports the hypothesis of a more pronounced immune mechanism.

Prematurity may further aggravate the clinical course of neonatal thrombocytopenia. Preterm neonates are more susceptible to bleeding complications due to immature hemostatic systems and increased vascular fragility, thereby increasing the risk of severe hemorrhagic events, including intracranial hemorrhage [4,8].

Another important differential diagnosis is neonatal alloimmune thrombocytopenia (NAIT), which is typically more severe and associated with a higher risk of intracranial hemorrhage compared with thrombocytopenia related to maternal ITP [9]. Distinguishing between these conditions is essential for appropriate management.

The assessment of fetal risk remains challenging in clinical practice. Invasive procedures such as fetal blood sampling carry significant risks and are not routinely recommended [9,10]. Currently, no maternal biological parameter has demonstrated sufficient predictive value for severe neonatal thrombocytopenia. The most reliable predictor remains a history of thrombocytopenia in a previous sibling [11].

Management of severe neonatal thrombocytopenia is well established. Current guidelines recommend the use of intravenous immunoglobulins (IVIG) and platelet transfusions in neonates with platelet counts below 30 G/L or in the presence of active bleeding [9]. In our case, the favorable response to IVIG supports an immune-mediated mechanism.

The prognosis of neonatal thrombocytopenia related to maternal ITP is generally favorable. Most cases resolve spontaneously within weeks to months, with a low risk of intracranial hemorrhage compared with alloimmune thrombocytopenia [8,12].

## Conclusions

This case highlights the potential severity of neonatal thrombocytopenia in infants born to mothers with severe ITP, particularly in those with a history of splenectomy. Early diagnosis, close monitoring, and multidisciplinary management are essential to prevent serious complications. Systematic platelet count assessment and careful clinical follow-up are strongly recommended in at-risk neonates.
